# Recent advances in the aqueous applications of PEDOT

**DOI:** 10.1039/d1na00748c

**Published:** 2021-12-01

**Authors:** Sam Rudd, Drew Evans

**Affiliations:** Future Industries Institute, University of South Australia Adelaide 5001 South Australia Australia drew.evans@unisa.edu.au

## Abstract

Water is ubiquitous in life – from making up the majority of the Earth's surface (by area) to over half of the human body (by weight). It stands to reason that materials are likely to contact water at some point during their lifetime. In the specific case of sensors however, there is a need to consider materials that display stable function while immersed in aqueous applications. This mini-review will discuss the most recent advances (2018 to 2021) in the application of the conducting polymer poly(3,4-ethylenedioxythiophene) (PEDOT) in aqueous environments. At its heart, the use of PEDOT in aqueous applications relies on nanoscale understanding and/or nanoengineered structures and properties. This enables their use in water-based settings such as within the human body or buried in agricultural soils.

## Abstract 2

The use of PEDOT in aqueous applications relies on nanoscale understanding and/or nanoengineered structures and properties. This enables their use in water-based settings such as within the human body or buried in agricultural soils.

## Graphical abstract

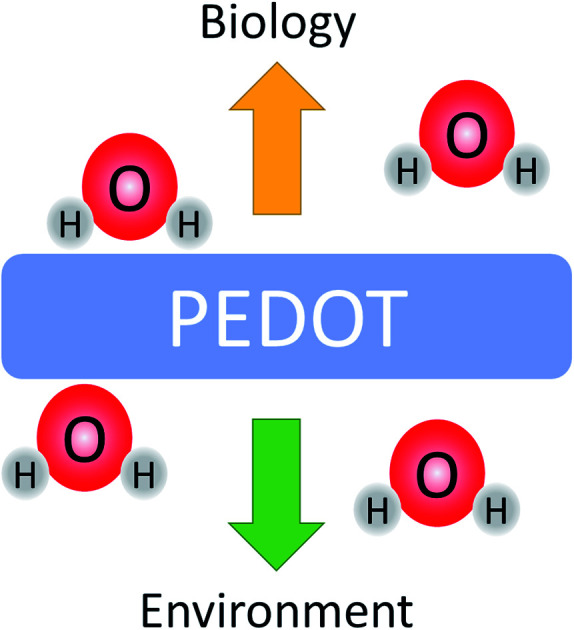

## 1. Introduction

There is currently a global effort to fabricate and utilise emerging nanomaterials, nanocomposites, and nanostructured materials with desirable characteristics for use in aqueous environments, such as biological fluids, aquatic ecosystems, and agricultural soil.^1–5^ The biocompatibility and sustainability aspects of the desired nanomaterials have been widely investigated with the aim of addressing concerns about how they behave in the mentioned environments.^6–9^ Subsequent to this, understanding and controlling the nanostructure and nanoscale dynamics of materials is also important to the devices integrating these materials. It is essential for the materials to be both nontoxic and robust, because in most water-based settings living organisms are found. At the intersection of these requirements is the search for materials that are biocompatible, stable, and functional (where function is dependent on the device operation). Conducting polymers (CPs) are one class of materials that have shown good compatibility with living organisms, good environmental stability, and excellent electrical function.^10–14^

Among all CPs, poly(3,4-ethylene dioxythiophene) (PEDOT) stands out as the prototypical CP displaying a range of desirable properties (relatively high electrical conductivity, high ambient stability, biocompatibility, tunable optical properties, *etc.*).^15–17^ The polymeric structure of PEDOT allows for electrostatic interaction with ions in the surrounding environment, making PEDOT an appropriate active material for sensor developments and controlled release drug delivery systems.^18–20^ Several recent studies have focused on understanding the interaction of PEDOT (doped with tosylate anions) with ions in water from a fundamental perspective. Delavari *et al.*^21^ computationally and experimentally studied the electrochemically driven ion exchange process in water. They showed that the PEDOT thickness increased upon repeated electrochemical cycling, indicative of water intake (facilitated by the hydration shell of the ions). This aligns well with recent work by Sethumadhavan *et al.* who experimentally observed the roll of water in the hydration shell around ions in the electrochemical reactions with PEDOT.^22^ They showed the classification of ions as structure-breaking or structure-making in water to be important for understanding how CPs interacts with ions in water.^23^ How these mechanisms on an atomic/molecular level impact on the deployment of PEDOT-based devices in water applications is of growing importance/interest. Therefore, this mini-review will present a brief overview of the recent key literature demonstrating the use of PEDOT in water-based applications. Focus will be placed on the nanoscale aspects of deploying PEDOT *in vitro* and *in vivo* for bioelectronics. Furthermore, this article also reviews the recent literature on the emerging applications of PEDOT in the environment (in the equivalent “environmental-friendly electronics”). For recent analysis of CPs for antifouling applications, which is an important consideration for their use in water, readers should refer to other reviews.^24–26^ Similarly, the fundamental properties of PEDOT have also been reviewed elsewhere.^15,27,28^

## 2. PEDOT in biology

PEDOT-based nanofilms/nanoparticles/nanocomposites represent a viable way to interface electronic devices with biological matter *in vitro* and *in vivo*.^29–40^ Routinely studied variants of PEDOT, such as those doped with PSS or tosylate, are promising materials for biological applications. Mokhtar *et al.*^37^ have investigated the stability of various doped PEDOT in biological fluid, *i.e.* artificial interstitial fluid (aISF), and demonstrated that PEDOT shows higher electrochemical stability in biological environments when it is doped with Tos or it is co-doped with Tos/PSS, as compared to doped with just PSS. Guzzo *et al.*^41^ have recently investigated the *in vitro* cytotoxicity of PEDOT:Nafion, and they have demonstrated that this form of PEDOT is not cytotoxic when it is prepared *via* water dispersion and an aqueous formulation, suggesting an alternative bioelectronic and neuroelectronic material for long-term applications such as chronic neural recording and stimulation sessions.

**Fig. 1:**
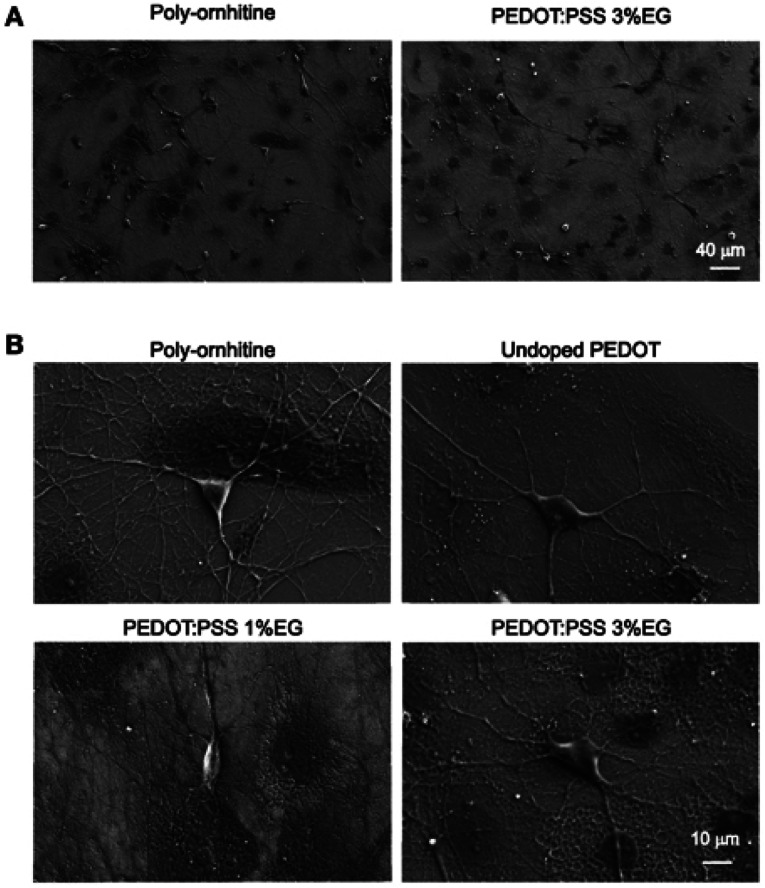
Scanning electron microscopy micrographs showing hippocampal cultures grown on PEDOT:PSS layers. (A) Lower magnification micrographs display that qualitatively the size of neuronal network is comparable between undoped PEDOT:PSS and PEDOT:PSS 3% EG. (B) Higher magnification micrographs show the healthy morphology of single neurons grown on the different substrates. Reproduced with permission from ref. 45. CC BY 4.0.

Despite the underlying biological performance of PEDOT, in some instances it may be necessary to modify it in some manner to improve its performance for biological applications. For instance, ethylene glycol (EG) based additives are seen as beneficial to improve the biocompatibility of the desired biological materials including PEDOT.^42,43^ Stříteský *et al.*^44^ used EG to modify PEDOT:poly(styrenesulfonate) (PEDOT:PSS) for biocompatibility testing of electroactive polymer inks for printed bioelectronics. The nanoscale CP layers (30 ± 5 nm) treated with EG served as a platform upon which to seed murine cardiomyocytes derived from embryonic stem cells. Along similar lines, Cellot *et al.*^45^ investigated the nanoscale morphology of PEDOT:PSS doped with different amount of EG (0.1–3%) for electrodes in neural applications. On a material level, for increasing EG content the nanoscale roughness (from AFM images) increased leading to an increase in exposed surface area up to 3% for 3% EG addition. They utilised the EG loaded PEDOT:PSS with hippocampal cultures to observe seeding and long-term proliferation of neuronal and glial cells, as highlighted in Fig. 1.

Poly(ethylene glycol)diglycidyl ether (PEGDE) is a derivative of poly(ethylene glycol) (PEG), which has been widely used for crosslinking of potential materials for biomedical applications.^46–49^ The research of Solazzo *et al.*^50^ introduced PEGDE to crosslink PEDOT:PSS for bio-applications. On a molecular level, the crosslinking occurs *via* the PEGDE epoxy ring interacting with the sulfonic groups of the PSS. Using CH310 mouse embryonic fibroblasts, they observed greater degrees of cell spreading on the crosslinked samples compared to the controls, *i.e.* PEDOT:PSS – glycidoxy propyltrimethoxysilane (GOPS); noting that pristine PEDOT:PSS could not be used as a control due to dissolution in the cell culture media. While the crosslinked PEDOT:PSS was quite hydrophilic (water contact angle < 20°) this alone couldn't explain the good biocompatibility. The introduction of the PEG moiety into the structure on the nanoscale was hypothesised as a key to improved biocompatibility.

In a recent review, Wang *et al.* discussed in detail the development of electrospun nanofibers using CPs for biosensors, neural electrodes, electrodes for stimulated tissue regeneration, and controlled drug delivery.^51^ This highlights that considering nanostructures of CPs such as PEDOT is beneficial for biological applications. Zhang *et al.*^52^ fabricated electrochemical polymerised PEDOT:PF_6_ with intertwined nanofibers. These nanostructured PEDOT materials displayed desirable electrical properties (×150 higher charge storage capacity, ×800 lower impedance, compared to the unmodified electrode) with appropriate *in vitro* biocompatibility and nontoxicity. This was demonstrated by assessing viability from culturing with PC12 cells, through to adhesion and differentiation of the same PC12 cells. Ultimately the nanostructured PEDOT led to formation of a network of neurites that were also longer and larger in number.^52^ An alternative method to achieve desirable nanostructure was presented by Richardson-Burns *et al.*^53^ with electropolymerised PEDOT:PSS around neurons which were subsequently removed using enzymatic and mechanical disruption. The creation of PEDOT:PSS with nanoscale cell-shaped holes and imprints showed good performance when re-seeded with SY5Y cells (which showed preference for adhering to regions where the neurons once were).

Opposed to nano-templating the PEDOT, Saunier *et al.*^54^ incorporated carbon nanofibers (CNFs) within PEDOT as microelectrodes for neuronal therapies. The PEDOT:CNF electrodes were used in electrochemical sensing of neurotransmitters (dopamine and serotonin). Furthermore, they showed *in vitro* non-cytotoxicity with SH-SY5Y cell populations having a viability percentage of >99%. In the work of Kumar *et al.*^55^ fluoro hydroxyapatite (FHA) nanoparticles were incorporated within PEDOT for use as coatings on implants. The introduction of the FHA nanoparticles led to subtle increases in the microscale roughness of the PEDOT with significant increase in the water contact angle and the mechanical hardness. These properties combined yielded a coating where the *in vitro* studies showed good adhesion of MG63 cells (human osteosarcoma cells) and increased levels of proliferation across 7 days of incubation. In implant scenarios, the antibacterial properties are equally important – with the PEDOT:FHA having significantly lower attachment and proliferation of both Gram-negative and -positive bacteria over a 24 h period, compared to the uncoated implant.

**Fig. 2:**
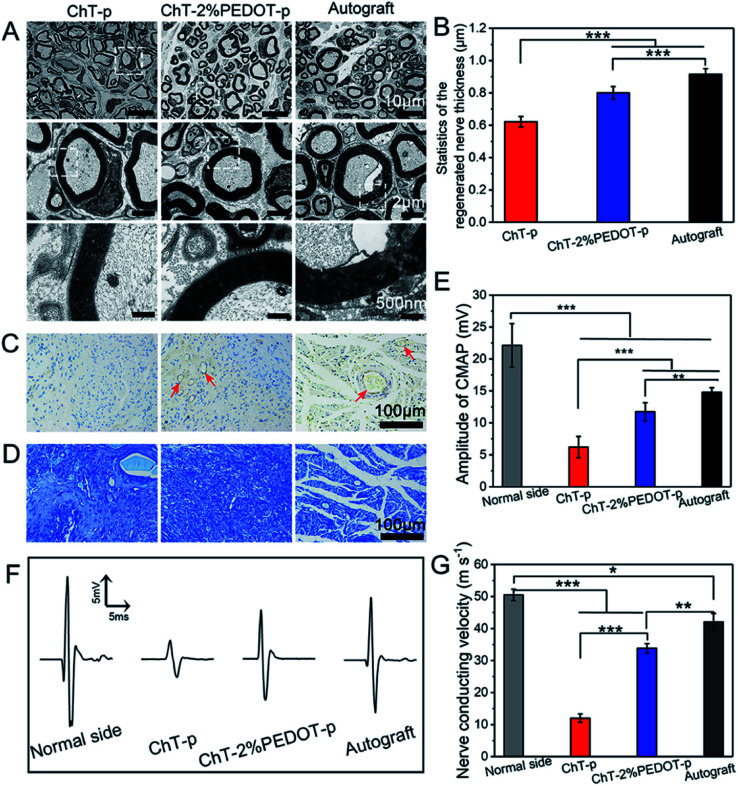
TEM images of the regenerated nerve (A), the thickness of the regenerated nerve (B) (standard error, *n* = 30, ***p < 0.001), immunohistochemistry staining of CD31 (C), and toluidine blue staining (D) of the regenerated nerve slice in chitin-p (ChT-p), chitin-2%PEDOT-p (ChT-PEDOT-p), and autograft group, respectively. The red arrow is indicated as vessels. The amplitude of compound motor action potential (CMAP) index of the normal side compared with experiment side (E). Nerve conducting velocity curve (F) and corresponded statistics (G) (standard error, *n* ≥ 3, ***p < 0.001, **p < 0.01, *p < 0.05). All testifications were conducted on the postoperative rats after 20 weeks. Reprinted with permission from ref. 56. Copyright 2021 American Chemical Society.

Rather than using PEDOT as the host matrix or major component of a composite/coating system, nanoparticles for embedding are also interesting for bioelectronics. Huang *et al.*^56^ have incorporated PEDOT nanoparticles into chitin hydrogels to improve sciatic nerve regeneration, by providing a desired physicochemical scaffold to promote the nerve cell proliferation. Chemical oxidative polymerisation was used to create PEDOT:persulfate nanoparticles of 200–300 nm in diameter. The porous structure of the hydrogel containing PEDOT nanoparticles has remarkably supported the enhanced *in vitro* RSC-96 cell adhesion. Once incorporated into the chitin hydrogel matrix and formed into a scaffold, the authors showed the benefit of the PEDOT nanoparticles through scaffold implantation in rat models. The *in vivo* assessment revealed that after 20 weeks post-surgery the chitin:PEDOT hydrogel had positively facilitated the sciatic nerve regeneration (Fig. 2).

Beyond presenting a favourable interface for desirable biological matter and/or an electrically conducting surface, PEDOT can also be repeatedly electrochemically doped and undoped in biological environments. Such behaviour makes PEDOT (and other CPs) promising materials for controlled/triggered drug delivery.^57–60^ Despite this promise, there are a relatively small number of research articles published in recent years on this topic. Yasin *et al.*^61^ employed the templating approach to form inverse opal structures of PEDOT:Tos using polystyrene nanospheres as a sacrificial support. The resultant 3D PEDOT material was used for loading and release of the model anionic drug dexamethasone phosphate (DexP^−^), which is used to treat an inactive/underactive adrenal gland or certain immune disorders and skin problems, asthma or arthritis. Through the creation of the 3D structured PEDOT the available surface area increased by 2.9 times above the unstructured form. Subsequently the passive loading of the DexP^−^ into the templated PEDOT was significantly higher (almost three times higher) and the films were more responsive to triggered drug release. Krukiewicz *et al.*^62^ incorporated DexP^−^ into electropolymerised PEDOT for enhancing neural growth. The porous and rough PEDOT structure lead to efficient triggered release of the DexP^−^ which had a positive effect on neurite growth. Woeppel *et al.*^63^ load sulfonate modified silica nanoparticles with electropolymerised PEDOT, and subsequently use these as reservoirs for two bioactive compounds (doxorubicin and melatonin). The PEDOT acted as an electrochemically responsive material that could load and release drugs, while the nanoparticle form factor allowed for delivery within a biological environment.

These studies combined highlight how PEDOT's good electrochemical properties can be combined with nanostructures or nanostructuring to yield benefits for biological systems – from biosensing to cell growth to drug delivery.

## 3. PEDOT in the environment

Water makes up a significant proportion of the world in which we live. These water environments are often critical to our existence, for example the agricultural soil where much of our food originates. It stands to reason that materials such as PEDOT have similar utility in these applications as they do in biology. This section briefly introduces the recent research using PEDOT in the environment – namely in plants, in soil, and in relation to contamination (and treatment) in water bodies.

**Fig. 3:**
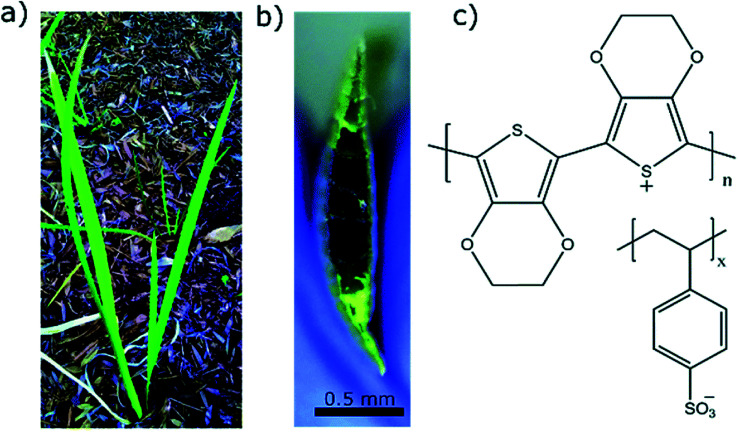
Examples of PEDOT used in plants. (a) Louisiana iris plant from which the leaf was extracted. (b) Cross sectional image of the leaf with PEDOT:PSS in the conduits which turns the conduits dark in color. (c) Chemical structure of PEDOT:PSS used as conducting polymer to construct conducting wires inside the leaves. Reproduced with permission from ref. 65. CC BY-NC 3.0.

Early works of Stavrinidou *et al.*^64^ demonstrated the concept of combining conducting polymers such as PEDOT with the xylem, leaves and veins in plants. Adhikari *et al.*^65^ recently injected PEDOT:PSS into the vascular channels of Louisiana iris leaves to create electrical capacitors (Fig. 3). Expanding on this, Kim *et al.*^66^ used vapour deposition to tattoo nanofilms of PEDOT onto the leaves of *Vitis vinifera* L. to monitor ozone oxidative damage. Impedance spectroscopy was used to interrogate the PEDOT electrode and make a statement about the ozone oxidative damage suffered by the leaves, as oxidative damage in plants changes the high-frequency impedance above 10^4^ Hz. Conversely, *bioristors*, organic electrochemical transistors for *in vivo* monitoring of key plant physiology parameters, were fabricated for insertion within the trunk of tomato vines^67^ and olive trees^68^ to assess changing function of the tree. The *bioristors* are comprised of commercial textile threads functionalised with EG treated PEDOT:PSS. These studies focused on measuring the *bioristors* resistance as a function of time and found strong correlation with plant transpiration, and postulate how mineral accumulation within leaves may be monitored. These hold promise for PEDOT, as a flora-compatible material, to enable live monitoring of growing plants.

In a similar manner, PEDOT may be used for live monitoring of agricultural soils – namely water and nutrients within the soil. Recently, nanocomposite films of PEDOT:PSS doped with a few wt% of titania nanoparticles (2–10 wt%) were used to determine the moisture content within model soils (montmorillonite and kaolinite).^69^ The resistance of the nanocomposite PEDOT:PSS displayed a linear response to changing soil moisture content, with a dependency on the specific soil type itself.

Typically contained within the soil water, nitrate (NO_3_^−^) is a key macronutrient in chemical fertilisers critical to plant growth and reproduction in modern agriculture, with overuse leading to contamination of groundwater and waterways, resulting in serious health, environmental and economic damage.^70^ Rudd *et al.*^71,72^ have explored the use of vapour deposited PEDOT:Tos for sensing of NO_3_^−^ in soil water. Interestingly the PEDOT nanofilms showed strong selectivity for NO_3_^−^ in the concentration range (1 to 100 ppm) typically used in agriculture for healthy plant growth. This was determined by measuring the electrical and optical property changes of the PEDOT nanofilms. Shahnia *et al.*^73^ leveraged this research to combine vapour deposited PEDOT nanofilms onto the end of optical fibres to detect NO_3_^−^ in water with a view to expanding the sensing range to NO_3_^−^ concentrations in aquatic ecosystems.

In some scenarios, ions in water present as an issue and define as undesired contaminants that need monitoring and/or removal. One ion that is related to overuse of fertiliser is nitrite (NO_2_^−^). Pang *et al.*^74^ functionalised PEDOT:PSS with silver nanoparticles to form a sensor for NO_2_^−^. In this study, the enhanced surface area from the nanoscale roughness combined with the specific surface chemistry was hypothesised to be the origins of the excellent sensing performance. Another class of chemicals that can contaminate water bodies are associated with fungicides and pesticides. In the work of Gao *et al.*^75^ they utilised PEDOT:PSS encapsulated within zinc metal–organic framework nanoparticles as an active material for electrochemical detection of dichlorophene. The PEDOT:PSS nanomaterial was able to detect the presence of dichlorophene dosed into lake water down to concentrations of 30 nM. Similarly, the fungicide Mancozeb (a dithiocarbamate biocide) was detected electrochemically using electropolymerised PEDOT that was functionalised with multiwalled carbon nanotubes and gold nanoparticles.^76^ The resultant composite thin film had roughness comparable to that of Pang *et al.* for sensing NO_2_^−^, with detection of the Mancozeb in the μM concentration range. These studies highlight that (nano)composites employing PEDOT, with their resultant nanoroughness and electrochemical properties, are useful in monitoring contaminants in water.

**Fig. 4:**
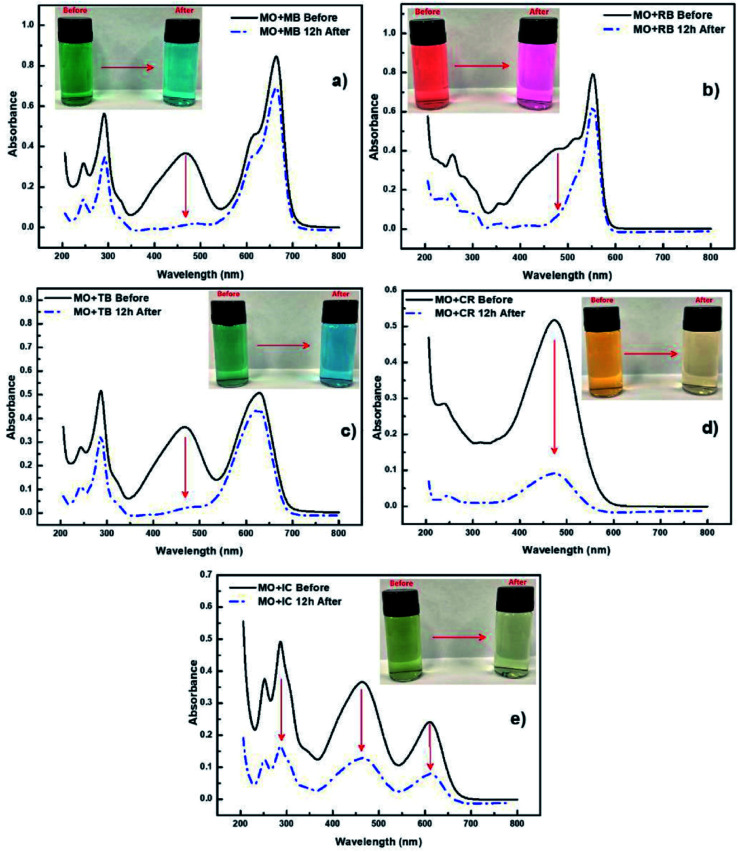
Selectivity experiments using cationic and anionic dyes accompanied by UV-vis spectra of (a) mixture of MO and MB dyes, (b) mixture of MO and RB dyes, (c) mixture of MO and TB dyes, (d) mixture of MO and CR dyes and (e) mixture of MO and IC dyes. Reprinted with permission from ref. 77. Copyright 2020 Elsevier.

Not only can chemicals be monitored, in some cases PEDOT can be used to assist in the removal and/or degradation of these chemicals. The removal of undesirable chemical components from contaminated water is important for producing water that is safe for a range of purposes, such as drinking, medical, and pharmaceutical applications. Methyl orange (MO) is one of the very common water-soluble azo dyes used in applications such as pH indicators, paper manufacturing, printings, food and pharmaceutical industries,^78^ causing shin eczema or intestinal cancer when is in contact with the skin or enters in the digestive system. da Silva *et al.*^77^ modified electrospun polymer fibres with PEDOT nanolayers to create a composite membrane for removal of MO from aqueous environments. They demonstrated the selectivity of the PVDF/PEDOT membrane to anionic dyes by exposing the membrane to a mixture of cationic and anionic dyes including MO (Fig. 4). This study showed that a shorter exposure time was required for the PEDOT modified membrane to interact with and absorb the MO and other anionic dyes (relative to cationic dyes) with at least 20 cycles of reusability, and operability over a wide pH range of 3–10. Pharmaceuticals are another example of undesirable chemicals found in water. For example, Metformin is a widely prescribed antidiabetic drug that can be found in reasonable concentrations in wastewater streams. Kumar *et al.*^79^ fabricated PEDOT powders that were used under UV light irradiation to photocatalytically degrade Metformin. These PEDOT (doped with Cl) powders were porous with an average size of 51 nm, leading to an active area of 1 m^2^ g^−1^, after use in the photocatalytic process. Like other studies discussed here, real water samples (secondary wastewater effluent) were spiked with the chemical of interest and the performance of PEDOT determined. Again, PEDOT showed good performance under these simulated conditions for degrading the model pharmaceutical drug.

## 4. Conclusions and perspectives

As discussed, nanoparticles, nanocomposites and nanofilms of PEDOT have gained recognition to be utilised in aqueous environments such as biology, agriculture and so forth. In particular, the fabrication versatility of PEDOT to form nanoscale materials combined with its electrochemical and biocompatibility properties in water-based environments make this polymer exciting for new applications such as biosensing. For example, PEDOT can be directly synthesised in the presence of living cells to bridge the biological signals and electronic processing systems, minimising the degree of foreign-body reaction of tissues. In addition, the improvement of reversible doping and de-doping phenomena in PEDOT has enhanced the design of nano-drug delivery systems with smart stimuli-responsive nanoplatforms. Furthermore, PEDOT possesses the promise of practical applications in a wide range of agricultural and environmental initiatives from chemical sensing to dye removal.

The outlook for future research should take into consideration pathways to commercial devices. This entails the scalability of the fabrication process to yield reproducible PEDOT nanomaterials at scale. Further to this is investigation of the long-term stability of PEDOT in water environments. Not only from a functional stability perspective but also from a safety, toxicity, and contamination perspective. This would allow for PEDOT to bridge the gap from lab-based research to deployment in biologically and/or environmentally relevant devices.

## Conflicts of interest

There are no conflicts to declare.

## Supplementary Material
